# *Mercury 4.0*: from visualization to analysis, design and prediction

**DOI:** 10.1107/S1600576719014092

**Published:** 2020-02-01

**Authors:** Clare F. Macrae, Ioana Sovago, Simon J. Cottrell, Peter T. A. Galek, Patrick McCabe, Elna Pidcock, Michael Platings, Greg P. Shields, Joanna S. Stevens, Matthew Towler, Peter A. Wood

**Affiliations:** a Cambridge Crystallographic Data Centre, 12 Union Road, Cambridge CB2 1EZ, UK

**Keywords:** *Mercury*, computer programs, crystal structure visualization, structure comparison, crystal packing

## Abstract

The program *Mercury*, developed at the Cambridge Crystallographic Data Centre, was originally designed primarily as a crystal structure visualization tool. Over the years the fields and scientific communities of chemical crystallography and crystal engineering have developed to require more advanced structural analysis software. *Mercury* has evolved alongside these scientific communities and is now a powerful analysis, design and prediction platform which goes a lot further than simple structure visualization.

## Abstract 2

An overview of *Mercury 4.0*, an analysis, design and prediction platform that acts as a hub for the entire Cambridge Structural Database software suite, is presented.

## 1. Introduction   

The program *Mercury* was first launched by the Cambridge Crystallographic Data Centre (CCDC) in 2001 as a focused crystal structure visualization tool. *Mercury* has since become established as a prominent crystal structure visualizer with a free-to-access version available for any researcher and many thousands of citations of its first two versions [at the time of writing 4608 for *Mercury 1.0* (Macrae *et al.*, 2006 ▸) and 5459 for *Mercury 2.0* (Macrae *et al.*, 2008 ▸)].

In the 18 years since the launch of *Mercury 1.0*, the fields of chemical crystallography (Watkin, 2010 ▸) and crystal engineering (Nangia & Desiraju, 2019 ▸) have changed dramatically, with much more sophisticated analysis of crystal structures now commonplace, alongside both knowledge-based and quantum chemical analysis of structures.

Over this time period, and very much driven by the requests of these communities, *Mercury* has become much more than a visualizer. *Mercury* is now a powerful platform delivering analysis, design and prediction functionality alongside visualization. The *Mercury* interface also acts as a hub for wider capabilities of the software suite built around the Cambridge Structural Database (CSD) (Allen, 2002 ▸; Groom *et al.*, 2016 ▸).

In the past decade in particular, the capabilities of *Mercury* have developed significantly, with a focus towards pharmaceutical and agrochemical solid-form informatics. These new components,^1^ collectively referred to as *CSD-Materials*, have been significantly driven by the CCDC’s industrial Crystal Form Consortium (https://www.ccdc.cam.ac.uk/Community/crystalformconsortium/), which was first introduced in August 2008. The discussions and direction provided by the CFC members over the years, which continue today, help to ensure both scientific value and clear industrial applicability in this area.

*Mercury* is used by a very broad community, both geographically and by subject area, and the CCDC continues to be directed by the needs of the scientific communities in how we plan the next iterations of the software. This paper in particular will illustrate the evolution of *Mercury* over the past decade from version 2.0, described by Macrae *et al.* (2008 ▸), up to the recently released version 4.0.

## 2. General *Mercury* functionality   

### 2.1. Ray-traced graphics   

**Figure 1:**
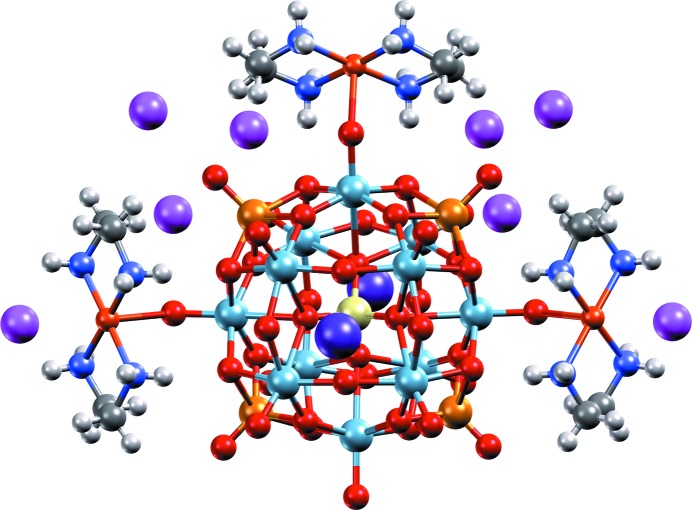
High-resolution image, generated with *Mercury* and *POV-Ray*, of a heteropolyoxoniobate-based system (CSD refcode LOFHOF; Zhang *et al.*, 2014 ▸) using the ‘Shiny’ material property.

The need to communicate science effectively continues to be a key challenge for scientists in outreach, education, research and industry. In order to make the generation of high-quality graphics and movies for science communication easier, *Mercury* now includes an intuitive interface to the ray-tracing rendering program *POV-Ray* (Persistence of Vision, 2013 ▸). This package, included in the *Mercury* installation, makes it straightforward to create high-resolution images (such as shown in Fig. 1 ▸) direct from *Mercury*, for use, for example, on journal covers, posters and presentations. Within the same interface, accessible through ‘POV-Ray Image’ under the ‘File’ menu in *Mercury*, movie frames of a rotating structure can also be quickly and easily generated, then combined together into a movie file.

### 2.2. 3D printing   

Science communication and education can also be aided by physical models representing structures. *Mercury* has recently been extended with functionality to generate 3D printable model files (in STL or VRML format) for experimentally accurate models directly from any standard 3D structural file format (including MOL2, XYZ, SDF, PDB, CIF and RES). The options, available under the ‘File’ menu using ‘Print in 3D’, include both colour (VRML) and monochrome (STL) 3D printing file output to provide compatibility with a wide range of existing 3D printers. There is also a lot of flexibility to control the size of the models and the thickness of all aspects of the models, including atom, bond and intermolecular contact thicknesses (Wood *et al.*, 2017 ▸).

### 2.3. Structure editing   

A range of structural editing features have been available for some time in *Mercury* (under the ‘Edit’ menu), which allow editing of bond types, editing of elements and addition of new atoms such as hydrogen atoms amongst a range of other options. Newer features are now included which allow the editing of the symmetry of a crystal structure as well as the unit-cell settings. Functions to transform molecules using the symmetry operators of the space group, as well as to apply inversion and translations, are also included. These new features can be helpful when comparing structures, as well as in preparing input files for quantum calculations.

**Figure 2:**
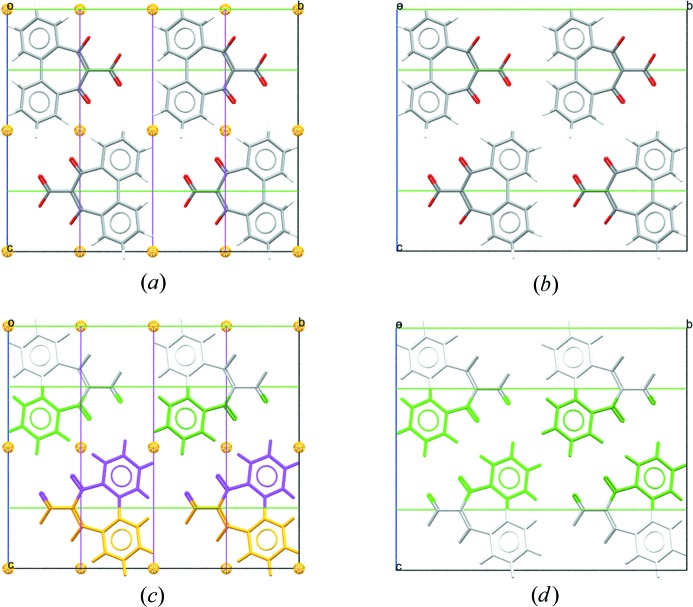
Packing diagrams for CSD refcode SUGCEC (Mochida *et al.*, 1992 ▸). (*a*) Unedited, presented in the original space group of *C*2/*c* with symmetry elements displayed. Note that the molecule lies on a twofold axis parallel to the *b* axis. (*b*) Unedited, with molecules coloured by symmetry operation (other than centring) and symmetry elements shown. (*c*) Edited to the subgroup *C*2, setting 1, origin choice [0, 0, 1/4], with symmetry elements displayed. (*d*) Edited to the subgroup *C*2, with molecules coloured by symmetry operation (other than centring). Transforming to *C*2 from space group *C*2/*c* retains only the twofold axes of the space group and increases the number of formula units in the asymmetric unit to two.

Different choices of space-group setting can now be explored, so a *P*2_1_/*c* structure with the *b* axis unique could be transformed to a *P*2_1_/*n* structure with the *c* axis unique, or a structure in *R* 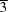 with hexagonal axes could be transformed to rhombohedral axes. Origin shifts can also be applied in each case. Going beyond changing settings within a space group, there are now options to change the space group entirely and allow the user to traverse the allowed maximal subgroups (as exemplified in Fig. 2 ▸). In each of these cases, only the allowable options are presented to the user, ensuring that the user can not unknowingly perform transformations that are not within the space-group definition provided by *International Tables for Crystallography*, Vol. A (2016 ▸).

### 2.4. Molecular shells   

Hydrogen bonds have been displayed in *Mercury* since the first release of the program, and hydrogen-bonding interactions from a central molecule to neighbouring molecules can be easily explored using the ‘expand hydrogen bond’ functionality. There has been no functionality introduced so far, though, that is specifically tailored to other types of interactions, such as π–π stacking. The ability to build and view the whole, or a subset, of a packing shell for a given molecule or part of a molecule is a useful tool for understanding of non-hydrogen-bonding interactions.

**Figure 3:**
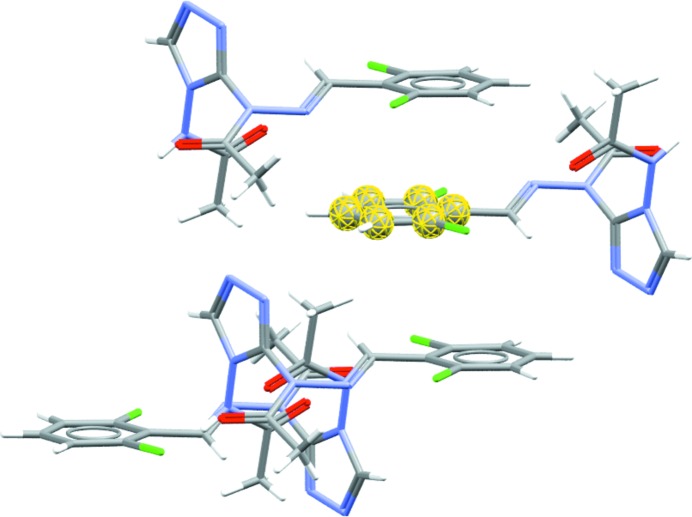
Diagram showing the molecular shell, calculated using a radius of van der Waals + 0.5 Å, from the phenyl ring fragment of a molecule in AABHTZ (Werner, 1976 ▸) (highlighted in yellow). The packing shell generated here highlights the aromatic interactions present in the structure. Full packing shells of molecules can be calculated by selecting a molecule (rather than a fragment) as the base unit.

To this end, it is now possible to calculate a molecular shell from a selected molecule, substructure or cluster of molecules using the ‘Molecular Shell’ option under the ‘Calculate’ menu. The neighbouring molecules that contain an atom within the user-specified distance from the selection are displayed (Fig. 3 ▸). Aromatic interactions can be explored, for example by selecting an aromatic ring in the base molecule and calculating a shell of molecules around the ring that extends to contacts within the sum of the van der Waals radii overlap + 0.5 Å. As an option to simply explore structures, there is also now a feature to simply click into space and reveal the closest symmetry-generated molecules to that area of the structure (‘Picking Mode’ > ‘Reveal Symmetry-Generated Molecules’).

## 3. *CSD-Community*   

A new menu has been introduced to *Mercury* including access to the components within *CSD-Community* – the suite of software and services provided free of charge by the CCDC for the benefit of the scientific community. From within *Mercury* it is now possible to link directly to the CSD web interfaces, allowing deposition and structural search of the CSD. There is also a special CSD Teaching Subset (https://www.ccdc.cam.ac.uk/Community/educationalresources/teaching-database/), as well as software for checking CIF syntax (Allen *et al.*, 2004 ▸) and unit-cell dimensions (*CellCheckCSD*; https://www.ccdc.cam.ac.uk/Community/CSD-Community/CellCheckCSD/). *CSD-Community* includes in addition a free version of *Mercury* incorporating a range of features for crystal structure visualization.

## 4. *CSD-System*   

The recently added ‘CSD-System’ menu in *Mercury* collects together links to the full set of *CSD-System* components, including *WebCSD*, *ConQuest*, Data Analysis, *Mogul* and *IsoStar*.

### 4.1. *WebCSD*   

*WebCSD* provides a web-based platform alongside the desktop *CSD-System* software. This web interface, covered under the normal CSD licence, provides new or occasional users with a simple and intuitive route to access the CSD, without the need to install any software locally. CSD structures can be accessed just by using a standard web browser on any computer, tablet or mobile device. Multiple search options are available, based on text queries, 2D chemical structure sketches, molecular formulae or unit-cell dimensions. Users can access the latest entries that have been added to the CSD on a continually up-to-date basis. *WebCSD* version 1 (Thomas *et al.*, 2010 ▸) was first launched in 2009, and in 2017 an updated version built on newer technologies was introduced (https://www.ccdc.cam.ac.uk/structures), which can be accessed via a menu item in *Mercury*.

### 4.2. Data search and analysis   

In previous versions of *Mercury* (Macrae *et al.*, 2008 ▸), structures and associated parameters from *ConQuest* searches (Bruno *et al.*, 2002 ▸) could be imported and viewed within *Mercury*. This has now been significantly extended through the ‘Data Analysis’ functionality in *Mercury*, providing a interactive interface connecting data analysis (spreadsheets, statistics and plotting of results) with 3D visualization of the structures (Sykes *et al.*, 2011 ▸). In this way, searches from *ConQuest* can be directly transferred into *Mercury* to analyse correlations or reveal statistically significant parameters within large data sets; alternatively, numerical data from a raw data file (.csv or .tsv) or a *CSD-Materials* packing feature search can be analysed.

In addition to ordering and filtering results through the data spreadsheet, the ‘Data Analysis functionality can calculate a variety of statistical descriptors for a distribution (such as the mean, variance, standard deviation and skewness), as well as creating new descriptors through arithmetic operations with the ‘Calculator’ functionality. It is also possible to carry out advanced analysis with correlation matrices and principal component analysis. A range of charting/plotting options is provided, including histograms, polar histograms, scatterplots (Cartesian/polar/heat) and heat maps. Subsets of the data can be easily created and highlighted to further explore trends. The plots are fully interactive with the 3D visualizer, allowing in-depth investigation of corresponding structures and their parameters.

**Figure 4:**
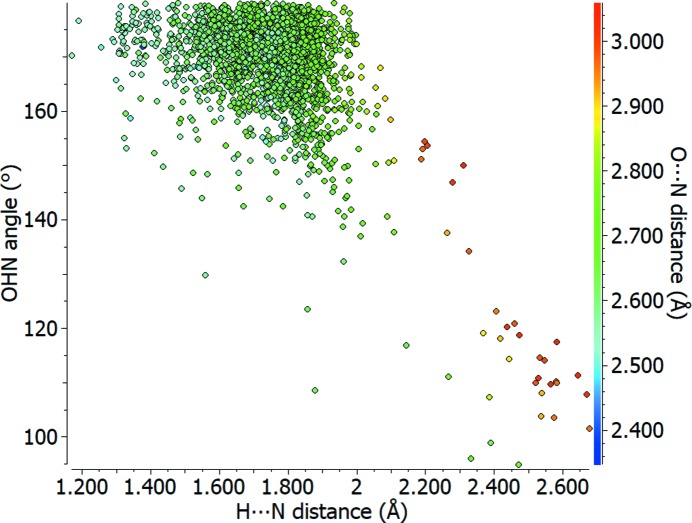
Scatterplot of O—H⋯N angle (°) against H⋯N distance (Å) for carb­oxy­lic acid to pyridine type nitro­gen–hydrogen bonds (O=C—OH⋯N=C), with the O⋯N distance (Å) shown using a colour scale.

An example of the output that can be generated is illustrated in Fig. 4 ▸, showing a coloured scatterplot of the hydrogen-bond angle (O—H⋯N) versus the hydrogen-to-acceptor distance (H⋯N) for a carb­oxy­lic acid donating to a pyridine-type nitro­gen, O=C—OH⋯N=C. The colour scale is used to show the donor-to-acceptor distance (O⋯N) as the third variable, with short distances in blue and longer distances in yellow to red. The greatest density of observed hydrogen bonds can be noticed in the region of 1.5–1.9 Å for the H⋯N distance and 160–180° for the O—H⋯N angle, dominated by green data points representing O⋯N distances of around 2.6–2.8 Å. Longer interactions (coloured yellow to red) are observed to have a greater spread in angle, with some of the longest contacts having angles close to 100°. The very shortest hydrogen bonds, ∼2.5 Å (blue), exhibit closer H⋯N distances as well as a tendency towards linearity, such that the hydrogen-to-acceptor and donor-to-hydrogen distances can become comparable in extreme cases. In these kinds of plots, the user can click on any point and immediately view the structure corresponding to that data point in the *Mercury* visualizer.

### 4.3. Knowledge-based analysis   

The CCDC’s two knowledge bases are key components of the *CSD-System* – these are *Mogul* (Bruno *et al.*, 2004 ▸) for derived intramolecular geometry data and *IsoStar* (Bruno *et al.*, 1997 ▸) for derived intermolecular interaction data. These quickly provide focused, chemically specific information on conformation and interactions because pre-derived libraries of relevant fragments already exist within the software. *Mercury* has had links to both of these knowledge bases since version 2.0, but these links have now been extended further to provide access to the desktop applications *Mogul* and *IsoStar* if the user wants to launch those components directly.

**Figure 5:**
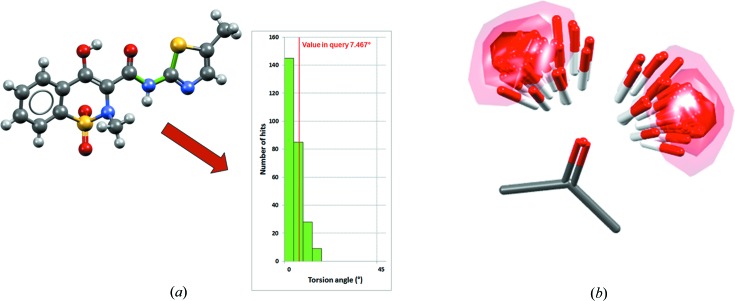
Illustration of CSD knowledge bases, showing (*a*) the *Mogul* distribution relating to a specific S—C—N—C torsion angle (bonds in the torsion are coloured green) in the meloxicam succinic acid co-crystal (CSD refcode ENICOU; Cheney *et al.*, 2010 ▸) and (*b*) the *IsoStar* scatterplot relating to a ketone central group interacting with an alcohol probe group (contact density shown as contour surfaces).

*Mogul* contains a hierarchical library of pre-derived distributions from the CSD of bond lengths, valence angles, torsion angles and ring conformations, allowing fast and chemically specific assessment of intramolecular geometry [see Fig. 5 ▸(*a*) for an example of a *Mogul* distribution]. *IsoStar* also contains pre-derived data, but in the form of scatterplots of intermolecular interaction distributions from which contour surfaces can be determined [see Fig. 5 ▸(*b*) for an example of an *IsoStar* scatterplot].

## 5. *CSD-Materials*   

*CSD-Materials* enables structural scientists to explore, analyse and design solid-state materials with the potential to understand structural stability, or explore new or modified solid-form properties. The components within *CSD-Materials* provide in-depth understanding of experimentally determined crystal structures as well as insight into the likely solid-form behaviour of new compounds. The capabilities include more sophisticated assessment of preferred intra- and intermolecular interactions, going beyond the scope of *Mogul* and *IsoStar* (‘Conformer Generator’ and ‘Full Interaction Maps’), as well as tools to help design new solid forms.

A collection of tools to help interpret and compare packing trends in crystal structures with CSD data using packing feature, similarity and motif searches (Wang *et al.*, 2014 ▸) was introduced in *Mercury 2.0* (Macrae *et al.*, 2008 ▸). Since version 2.0, the quality of structural data and continued expansion in size of the CSD, as well as demand from industrial users, has allowed the development of further sophisticated statistical techniques harnessing this information. In this vein, data-driven tools for prediction and analysis have been developed in *CSD-Materials*, such as automated polymorph risk assessment via hydrogen-bond propensity (Majumder *et al.*, 2013 ▸; Feeder *et al.*, 2015 ▸), co-crystal screening (Sandhu *et al.*, 2018 ▸) and the conformer generator, which allows geometry exploration. In addition to these tools, new components have recently been introduced to allow the user to analyse complex solvate and hydrate structures (see Sections 5.3.2 and 5.3.3).

### 5.1. Polymorph assessment   

A knowledge-based method has been developed to assess the risk of polymorphism based on hydrogen bonding. This methodology applies statistical analysis using logistic regression and is trained against observed hydrogen bonds in the CSD (Galek *et al.*, 2007 ▸). It results in the generation of all possible hydrogen-bonding networks for a given system, with knowledge-based assessment of the likelihood of each possible network. This helps significantly in the assessment of a given crystalline form and is usually applied alongside other analytical CSD-based techniques (such as conformational analysis and full interaction maps) in a solid-form risk assessment.

**Figure 6:**
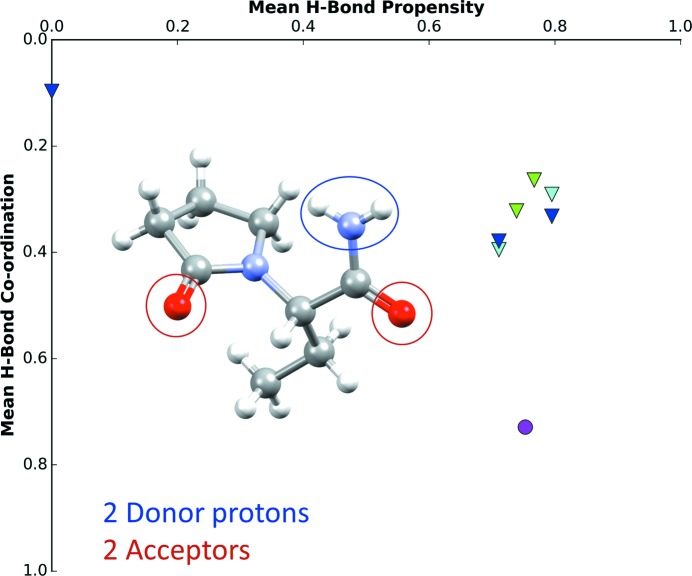
Hydrogen-bond propensity chart of levetiracetam (CSD refcode OMIVUB; Song *et al.*, 2003 ▸). The magenta circle represents the observed structure.

For example, levetiracetam (CSD refcode OMIVUB) is a monomorphic system with two potential proton donors and two acceptor functional groups (Fig. 6 ▸). The hydrogen-bond propensity chart displays all possible hydrogen-bond networks, with the most likely hydrogen-bond network displayed in the lower-right corner. The observed structure is represented as a magenta circle. In this case, the observed network is ranked competitively in terms of hydrogen-bond propensity and it utilizes the functional groups optimally, as demonstrated by the high mean hydrogen-bond coordination score.

### 5.2. Co-crystal design   

Co-crystals have represented a significant area of interest and development over recent years, with huge potential for modifying and even tailoring physicochemical properties of a target molecule by co-crystallizing with a second molecular component (co-former). Considering the potential range of co-formers, a knowledge-based approach to co-crystal design is extremely valuable for assessing the likelihood of co-crystal formation (Wood *et al.*, 2014 ▸). This can be considered in two steps: (1) the screening out of co-formers to remove those highly unlikely to yield a co-crystal with the target molecule, and (2) subsequent ranking of the more likely co-former candidates.

(1) *Mercury* now incorporates virtual co-crystal screening through the ‘Molecular Complementarity’ component under the ‘Co-Crystal Design’ menu within *CSD-Materials*. This is based on calculated quantitative structure–activity relationship molecular descriptors (Fábián, 2009 ▸; Galek *et al.*, 2007 ▸, 2009 ▸, 2010 ▸), whereby molecules that are observed to co-crystallize tend to have similar properties (*e.g.* shape, polarity). Threshold values for the molecular descriptors have been defined, on the basis that the majority of co-crystal entries in the CSD (90%) have been assessed as likely to co-crystallize (Fábián, 2009 ▸). Therefore these thresholds allow co-formers that are unlikely to be effective for the target molecule to be screened out prior to starting experimental screening. Assessment is performed for a chosen library of possible co-formers against a range of different conformations for the target molecule (user-specified or generated using the ‘Conformer Generation’ tool).

Molecular complementarity analysis has been found to be particularly effective in cases where co-crystal formation is difficult. In the case of artemisinin only two out of 75 co-formers were successful in experimental screening, but use of mol­ecular complementarity analysis would have ruled out 33 of those 75 co-formers (44%), boosting the success rate of experiments and reducing the time spent (Karki *et al.*, 2010 ▸). Details of the co-crystal screening results can also be exported, allowing further investigation: for example, whether co-formers with low hit rates only pass with a specific conformation of the target molecule.

(2) Once the least likely co-formers have been excluded, *Mercury* can be used to assist with ranking of co-formers that have passed the first screening step; this can allow optimization of co-crystallization experiments by trying the co-formers that are most likely to be effective first. (i) The ‘Motif Search’ component (Macrae *et al.*, 2008 ▸) reveals the frequency of occurrence for different motifs involving functional groups of a target molecule. This can be utilized to probe which co-formers contain motifs that are most commonly observed and thus have a higher likelihood of being successful in co-crystal formation. (ii) The ‘Hydrogen Bond Propensities’ tool within *Mercury* (see Section 5.1) evaluates the likelihoods of all possible hydrogen-bond interactions between molecules, taking into account parameters such as competition and steric hindrance (Fábián, 2009 ▸; Galek *et al.*, 2007 ▸, 2009 ▸, 2010 ▸). This can be used to compare the best hetero- and homo-hydrogen-bond interactions between the target molecule/co-former pair, with a higher difference between the two indicating a stronger hydrogen-bond-based drive towards co-crystallization.

### 5.3. Structure analysis   

#### 5.3.1. Full interaction maps   

A complementary approach to analyse structures and rationalize the stability of polymorphs has been introduced in *Mercury* through visualization of knowledge-based interaction maps around molecules in a crystal structure (Wood *et al.*, 2013 ▸). The ‘Full Interaction Maps’ component within *CSD-Materials* can be used to evaluate the preferred interactions of a molecule (Honorato *et al.*, 2019 ▸; Price *et al.*, 2014 ▸).

**Figure 7:**
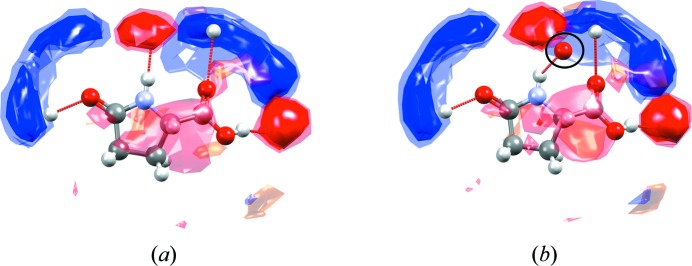
Full interaction maps shown around one of the molecules of l-pyroglutamic acid in (*a*) the (stable) α form (CSD refcode LPYGLU07, *Z*′ = 3) and (*b*) the (metastable) β form of the compound (CSD refcode LPYGLU08, *Z*′ = 1) (Panda *et al.*, 2015 ▸). The circled oxygen acceptor in the interaction map diagram shown for the β form (*b*) is observed outside of any preferred acceptor region of the maps (red contours), indicating that this hydrogen-bonding interaction has a sub-optimal geometry.

For example, Fig. 7 ▸ illustrates the full interaction maps for a crystal that exhibits jumping properties, l-pyroglutamic acid, before and after a phase transition. The blue regions of the interaction maps highlight where donors are expected to be observed on the basis of CSD data, the red regions indicate likely acceptor positions and orange regions indicate likely positions of hydro­phobic groups. The interaction maps are scaled relative to the random chance of that particular contact occurring, so the standard contour surface levels of 2, 4 and 6 (with increasing levels of opacity) indicate 2, 4 and 6 times greater interaction likelihood than random chance, respectively.

Upon heating of l-pyroglutamic acid, crystals of the α form [Fig. 7 ▸(*a*); only one of the unique molecules is shown as an example; *Z*′ = 3] convert to the β form [Fig. 7 ▸(*b*)], and this phase transition is observed to induce a jumping motion of the crystalline material (Panda *et al.*, 2015 ▸). In both structures of l-pyroglutamic acid, full interaction maps show four well defined hotspots with some additional diffuse interaction regions. In the β form [Fig. 7 ▸(*b*)], one of the observed hydrogen-bonding interactions (involving the O atom of the carb­oxy­lic group, circled) lies well outside the expected area of the relevant acceptor hotspot, indicating that the geometry of this particular interaction is sub-optimal compared with what would be expected from CSD data. The full interaction maps in the β form (metastable at ambient conditions) are therefore not fully satisfied. In contrast, the key hotspots in the full interactions maps for the α form (stable at ambient conditions) are each satisfied by an interaction.

#### 5.3.2. Hydrate Analyser   

The ‘Hydrate Analyser’ component within *CSD-Materials* provides a quick and powerful approach to analyse even the most complex hydrated structures. This feature provides the capability for the user to investigate interactions formed by water as well as to visualize the space taken up by the water molecule. The hydrogen-bond interactions displayed by the water molecule are automatically classified on the basis of the ten most common water hydrogen-bonding motifs found in the CSD (Gillon *et al.*, 2003 ▸). The user can identify the hydrogen-bonding motifs by clicking on the motif in the dialogue, which results in the display of the chosen interaction in the 3D visualizer of *Mercury*.

**Figure 8:**
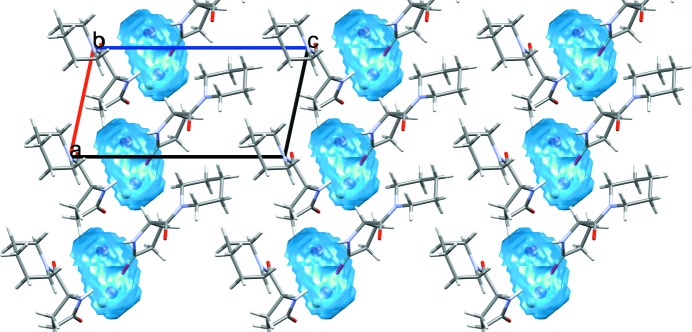
Calculated water space for fasoracetam monohydrate (CSD refcode PAPNIG; Harmsen *et al.*, 2017 ▸) with water molecules occupying discrete pockets within the crystal structure.

The volume occupied by the water molecule can be calculated under the ‘Water Space’ tab. The calculation is performed using the same method as the established ‘Voids’ feature in *Mercury*. This functionality can be used to display the volume and shape of the space occupied by water in a known hydrate, as well as to identify the possible presence of water in a crystal structure containing voids. The default probe radius is set to 1.2 Å, which is the approximate molecular radius of a water molecule. The user can visualize the water behaviour within the crystal structure, whether it is occupying discrete pockets, as in the example displayed in Fig. 8 ▸, or is forming channels.

Under the ‘Water Interaction Map’ tab, the user can assess whether the water molecules are occupying the location that is expected on the basis of the interaction maps. This feature allows calculation of interaction maps around the molecules in the structure based on a specific water probe.

#### 5.3.3. Solvate Analyser   

Similar to the ‘Hydrate Analyser’, a ‘Solvate Analyser’ component is now available within *CSD-Materials*. This feature enables a fast analysis of complex solvate structures, including those with more than one solvent, a mixture of solvents, co-formers and counter-ions, and even highly disordered solvate structures. Like in the ‘Hydrate Analyser’, the space occupied by the solvent molecules is calculated using the same method as is used for calculating voids. The tool allows the user to select solvents from the displayed structure and calculate the space occupied by those molecules. A summary of the hydrogen-bonding motifs displayed by the solvent molecules is listed under the ‘Solvent H-Bonding’ tab.

**Figure 9:**
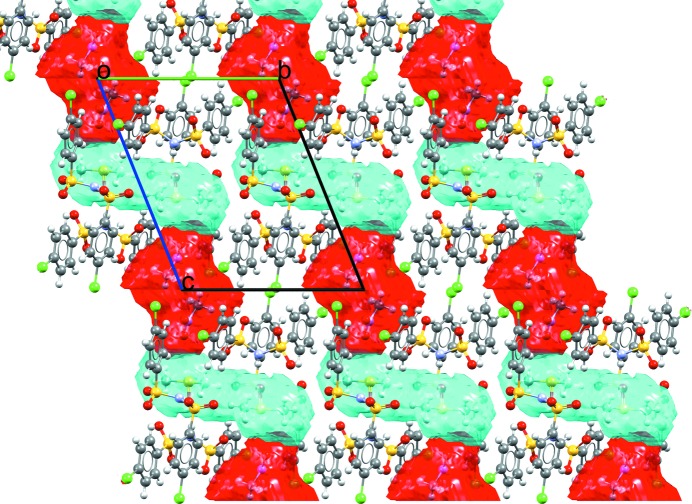
Calculated solvent space for bis­[bis­(4-chloro­benzene­sulfonyl)­amine] di­methyl­sulfoxide (space shown in cyan) nitro­methane (space shown in red) solvate (CSD refcode XUKZIM; Hamann *et al.*, 2002 ▸).

CSD refcode XUKZIM is an example of a complex solvated structure containing a mixture of solvents: di­methyl­sulfoxide and nitro­methane. The space occupied by the solvents within the crystal structure can be calculated using the ‘Solvate Analyser’ and visualized; di­methyl­sulfoxide and nitro­methane form *S*-shaped channels along the crystallographic *c* axis (Fig. 9 ▸).

### 5.4. Conformer Generation   

**Figure 10:**
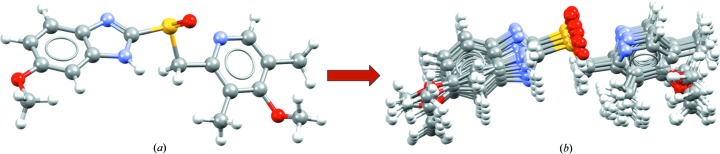
Conformer generation for the molecule omeprazole, starting from (*a*) the molecular conformation in a known crystal structure (CSD refocde VAYXOI; Ohishi *et al.*, 1989 ▸) and generating (*b*) a diverse conformer ensemble including the 15 highest ranked conformers.

The ‘Conformer Generator’ component uses CSD-derived rotamer and ring geometry information in order to minimize the conformation of a molecule or generate a diverse set of high-likelihood conformers for a given input molecule. Energy is not explicitly used as a criterion in the CSD Conformer Generator (Cole *et al.*, 2018 ▸), rather a statistical appreciation of high and low probability conformations based on CSD knowledge. A diverse set of likely conformations for a mol­ecule can be useful for *in silico* co-crystal screening as well as for ligand-based or structure-based drug design endeavours. An initial 3D conformation of a target molecule is provided as input, and from this, a diverse range of conformations can be generated, with control of the number of conformers produced (Fig. 10 ▸). More information about the methodology used to generate and rank conformations can be found a recent paper by Cole *et al.* (2018 ▸).

### 5.5. Structure solution from powder data   

Crystal structure solution from powder diffraction data can be performed using the program *DASH* (David *et al.*, 2006 ▸), another component within *CSD-Materials*. Structures can be solved using simulated annealing approaches within *DASH*, and the success rate can also be improved by reducing conformational search space using CSD data (*Mogul*). The tool has been successfully used to solve structures from various fields including pharmaceutical systems (*e.g.* piroxicam; Naelapää *et al.*, 2012 ▸), salts (*e.g.* tenapanor dichloride; Nilsson Lill *et al.*, 2018 ▸), solvates (*e.g.* darunavir ethanol; Kaduk *et al.*, 2015 ▸), semiconductors (*e.g.* lithium 1,8,15,22-tetra­phen­oxy­phthalocyanine; Pandian *et al.*, 2007 ▸) and explosives (*e.g.* silver azide; Schmidt *et al.*, 2007 ▸).

Structure solution from powder data in *DASH* still requires good-quality high-resolution powder data to be collected and a single phase to be present in the diffraction pattern, but structures with over 20 degrees of freedom can now be routinely tackled. Two recent studies have shown the benefits of using *Mogul* torsion data as well as optimized simulated annealing parameters in *DASH*, resulting in impressive increases in success rates (Kabova, Cole, Korb, López-Ibáñez *et al.*, 2017 ▸; Kabova, Cole, Korb, Williams & Shankland, 2017 ▸). Other researchers have previously published detailed recommendations on how to maximize the chances of success of structure solution from powder X-ray diffraction data by collecting high-quality data sets (Florence *et al.*, 2005 ▸).

### 5.6. Calculations   

#### 5.6.1. *MOPAC*   

**Figure 11:**
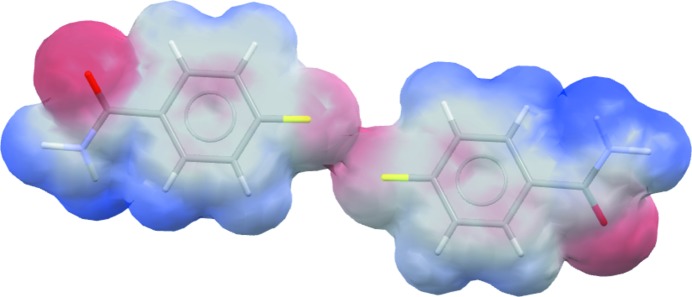
The electrostatic potential mapped onto the van der Waals surface of 4-fluoro­benzamide (CSD refcode BENAFP; Takaki *et al.*, 1965 ▸), illustrating the repulsive nature of the F⋯F contact (centre).

Options such as geometry optimization, assignment of bond orders and calculation of electrostatic potential are now available within the ‘MOPAC’ component of *CSD-Materials*, which links to the standalone *MOPAC* program (Stewart, 2016 ▸). The *MOPAC* interface in *Mercury* allows the user to run calculations using several Hamiltonians, such as AM1, MNDO, MNDOD, PM3 PM6, PM7 and RM1. The electrostatic potential mapped onto van der Waals surfaces can, for example, be used to investigate non-covalent interactions such as in the case of the pair of molecules illustrated in Fig. 11 ▸ involving an F⋯F close contact in 4-fluoro­benzamide (CSD refcode BENAFP). The fluorine atoms are shown to have a negative electrostatic potential on the surface (red), suggesting that the observed F⋯F contact in this case is repulsive.

#### 5.6.2. Intermolecular energies   

Force-field-based intermolecular energy calculations can be performed using the ‘UNI Intermolecular Potentials’ component (Gavezzotti, 1994 ▸; Gavezzotti & Filippini, 1994 ▸) within *CSD-Materials*. These calculations are quite approximate in nature as they use empirical pair-potential parameters, but they allow the user to quickly assess the relative influence of the dimers in the structure, including the likely effects of different hydrogen-bonding interactions, aromatic interactions and other contacts.

**Figure 12:**
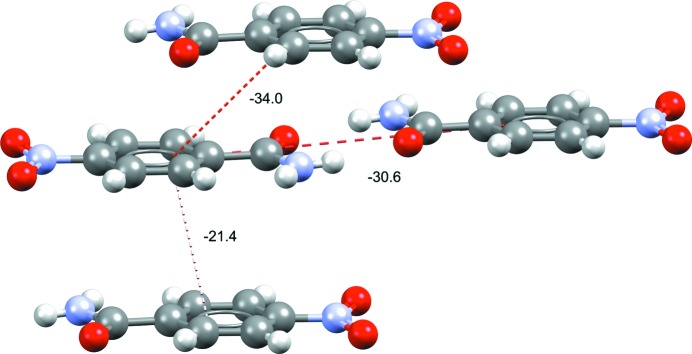
Dimer intermolecular interaction energies (kJ mol^−1^) of the top three interaction energies in form I of 4-nitro­benzamide (refcode NTBZAM10; Di Rienzo *et al.*, 1977 ▸). Stacking-related dimers are seen to be close in interaction energy to the observed hydrogen-bonded dimers.

This interface allows the calculation and display of interaction energies between molecules within the 3D visualizer of *Mercury* using a range of display options. The number of interactions to display can be tailored by the user, and there are options to customize the interaction line colour, or thickness, by the interaction energy. Fig. 12 ▸ shows an example where dimer energies are displayed for the structure of nitro­benzamide. Despite the observation of very clear hydrogen bonding in the structure, it can quickly be seen that stacking interactions are likely to also be important in the stability of this structure.

## 6. *CSD-Discovery*   

*CSD-Discovery* provides a suite of tools developed to aid computational and medicinal chemists in designing new active molecules by gaining insights from high-quality crystal structure information. Within *Mercury*, a collection of links to key components are gathered under the *CSD-Discovery* menu item, enabling access to a range of tools for exploration and knowledge building.

The components in *CSD-Discovery* can aid both structure-based and ligand-based drug design. Within *Mercury* itself, users can perform analysis of full interaction maps for mol­ecules to better understand their interaction preferences, and the generation of conformations based on CSD-derived data can facilitate computer-aided drug design efforts. Also in the area of structure-based drug design, from *Mercury* users can launch protein-ligand docking analyses through *GOLD* (Jones *et al.*, 1997 ▸) and perform the mapping of interaction hotspots from protein cavities using *SuperStar* (Verdonk *et al.*, 2001 ▸). If the structure of the target protein is not available, then the intelligent overlay of ligands (Taylor *et al.*, 2012 ▸) which bind to the target protein can be used to assess the key interactions and help to predict new active molecules.

## 7. CSD Python API   

**Figure 13:**
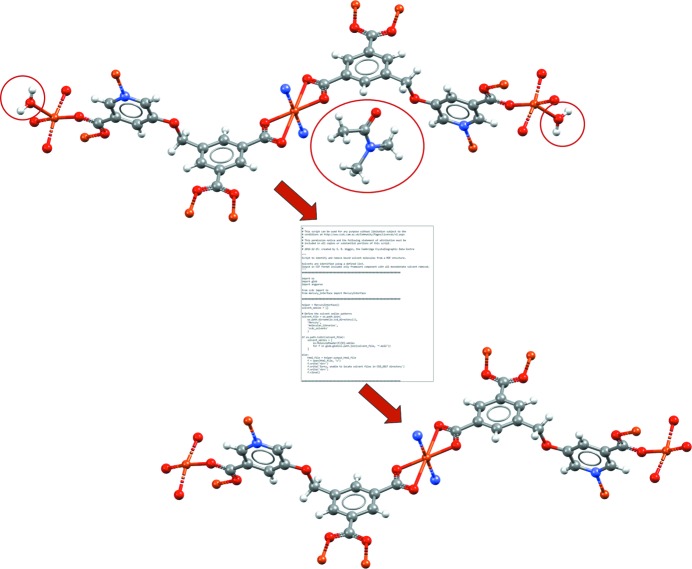
A CSD Python API script that can be run within *Mercury*. This example shows a script allowing automated removal of both unbound and bound solvent molecules (matching to a specified solvent library) from a MOF structure. The example shown is of a copper MOF structure (CSD refcode BEXSII; Patra *et al.*, 2003 ▸). In this case the Python script takes the unedited crystal structure as input (top), removes the unbound dimethylformamide solvent (circled) as well as the two singly bound water molecules (circled) and outputs the edited crystal structure for viewing in *Mercury* (bottom).

*Mercury* now provides a flexible interface allowing users to run tailored Python scripts within the program, making use of the CSD Python application programming interface (API). The CSD Python API allows a wide range of structural analyses available from all the various user interfaces (including *Mercury*, *ConQuest*, *Mogul*, *IsoStar* and *WebCSD*), as well as access to features that are currently inaccessible through any of the graphical interfaces. A number of scripts are made available within *Mercury* as examples. These are easy to customize and apply for specific scientific studies, providing ways to produce bespoke reports or specific analyses, like similarity searches. A more complex example was published by Moghadam *et al.* (2017 ▸), who wrote a Python script to take any metal–organic framework (MOF) structure in *Mercury* and automatically remove both unbound and bound solvent molecules for easier analysis or subsequent calculations (Fig. 13 ▸).

The CSD Python API script menu within *Mercury* allows any structural file formats loaded within the *Mercury* visualizer to be used as input, as well as text inputs; outputs can be easily read into the *Mercury* interface in the form of new/edited structural files, reports or spreadsheets of data.

## 8. Documentation, availability and environment   

*Mercury* has an extensive user guide with several tutorials available. Documentation can be accessed either through the program or via the CCDC web site (https://www.ccdc.cam.ac.uk). A standalone version of the program is available from the *Mercury* section of the CCDC web site (https://www.ccdc.cam.ac.uk/mercury/) and can be obtained for use as a visualizer by anyone worldwide, though some of the features require a CSD licence. The new functionality described in this publication is spread across the different CSD licence levels, with Sections 2.1, 2.2 and 3 relating to freely available features and the rest requiring some form of CSD licence.

The CSD software is kept up to date with the latest operating systems across Windows, Linux and macOS. As of version 4.0, released in the 2019 CSD Release, *Mercury* is supported on a range of platforms, including Windows (Intel compatible, 32-bit executables: Windows 7 and 10), Linux (Intel compatible, 64-bit executables: RedHat Enterprise 6 and 7, CentOS 6 and 7, Ubuntu 16) and macOS (Intel compatible, 64-bit executables: 10.12, 10.13, 10.14). Note that the Windows executables are compatible with both 32-bit and 64-bit versions of Windows.

## 9. Conclusions   

Not only is the program *Mercury* an effective crystal structure visualizer, but it has also evolved to become an advanced analysis, design and prediction platform. Here, we describe the capabilities of version 4.0 of *Mercury*, particularly charting the development of the software over the past decade since version 2.0 in 2008. The program has always been closely linked to the fields of chemical crystallography and crystal engineering, evolving quickly over recent decades, just as the science in those fields has done. These communities will continue to play a key role in defining the future directions of *Mercury*.

## Footnotes

1 The 13 components available in *CSD-Materials* at the time of writing are as follows: Motif Search, Crystal Packing Feature Search, Crystal Packing Similarity, BFDH Morphology, MOPAC Calculations, UNI Intermolecular Potentials, Hydrogen Bond Propensity, Co-Crystal Design by Molecular Complementarity, Full Interaction Maps, Hydrate Analyser, Solvate Analyser, Conformer Generator and DASH. The original three components available in the *Materials Module* of *Mercury* in 2008 were Motif Search, Crystal Packing Feature Search and Crystal Packing Similarity.
